# The Antiseptic Instinct

**Published:** 1899-11-11

**Authors:** 


### The Antiseptic Instinct.

The severe onslaught recently made by Professor Ogston upon the position, the training, and the general capacity of the officers of the Royal Army Medical Corps has been regarded from many points of view, and among the various comments which have been made on the subject, some have been by no means complimentary to the Army Medical Service. Under these circumstances it is perhaps unfortunate that the very wise decision of the War Office to send out to South Africa a considerable number of civil surgeons, and to institute a consultative body with the President of the Royal College of Surgeons at its head, should have appeared to lend some support to the allegations which have been so freely made in regard to the inefficiency of our present Army Medical Service. It may be well then to point out that the appointment of Sir William MacCormac and those who have gone out with him in a consultative capacity was not meant as nor did it entail any slur upon the Royal Army Medical Corps. However high a man may stand in his profession, there can be nothing derogatory in being asked to consult in serious cases with such men as the British Government has sent out to the assistance of its wounded soldiers in South Africa. This is a point which neither needs nor bears any discussion. The Government has endeavoured to provide the best surgical talent for the succour of the wounded, and in so doing it has done well. But a considerable number of men are being sent out who are not of consultant rank, and in regard to them it is being asked why should they be sent to the exclusion of army men, and more especially of army medical officers who have retired on gratuity but still remain under a liability to be recalled to service. These form a sort of Medical Reserve, and the fact that young house surgeons are being sent out, while the services of these retired medical officers have not been accepted, is likely, unless explained, to be taken as bearing out the evil things that have been said against the Army Medical Service. In regard to this, then, it has to be pointed out that it is not a case of merely sending out "more surgeons" but of supplying the base hospitals, and perhaps even those nearer to the front, with all the appliances which civil hospitals enjoy—including surgeons thoroughly accustomed to operative work and thoroughly trained in modern operative technique. Such men are chiefly to be found among the junior staffs of our great hospitals, where they have become completely imbued with the antiseptic instinct. We would lay especial stress upon the latter, for it is the great and, perhaps, the most characteristic trait by which the hospital surgeon who is always operating stands apart from the surgeon who only operates occasionally. The knowledge of each may be the same, but, while the occasional surgeon has to think, and think carefully, about all those details in regard to the maintenance of asepsis on which success depends, to the hospital surgeon these things have become a sort of instinct. Mr. Brudenell Carter, in a letter to the *Times*, has well put this matter before the public. He points out that it may make the difference between life and death, or the difference between recovery and mutilation, to hundreds of brave men, whether their injuries receive the sort of attention which would now be given to them in a London hospital, or that which is the best reasonably to be expected from operators of slender and restricted experience. In order to obtain success an operation must be conducted with perpetual reference to a multitude of precautions as to matters of minute detail—precautions apparently trivial or even superfluous, and, what is more, often actually superfluous. But in the midst of an operation there is neither time nor opportunity to discuss or to decide whether this or that precaution against septic infection shall be taken. We must do more than what may be absolutely necessary. By constant practice these precautions become a second nature, and it is by the cooperation of people who have thus attained the instinct of using aseptic methods that the success of the operative work in our hospitals is largely due. As Mr. Carter says:—

> "These precautions, on which the exclusion of disease-producing bacteria depends, become second nature, or a sort of acquired instinct to the operator who is always observing them, but are sadly likely to be in some point overlooked or neglected, especially under the pressure of great emergency, by the operator who has indeed read about them and whose theoretical knowledge may be complete, but whose opportunities of practice have been insufficient to render them living realities to his mind. The difference is analogous to that which exists between a language spoken by a native and the same language spoken by a foreigner. With the former correctness is instinctive, with the latter occasional errors are inevitable."

There is no doubt that all our military surgeons with the general surgical knowledge they possess would soon become also possessed of this instinct of asepsis. But our soldiers are there now, waiting for the best that can be given them; and if we want this instinct of asepsis, which is the key to success in operative surgery, we must get it ready made, as it exists in those who are at work in our great hospitals.

